# Seroprevalence and epidemiology of hepatitis B and C viruses in pregnant women in Spain. Risk factors for vertical transmission

**DOI:** 10.1371/journal.pone.0233528

**Published:** 2020-05-21

**Authors:** Ángeles Ruiz-Extremera, María del Mar Díaz-Alcázar, José Antonio Muñoz-Gámez, Marta Cabrera-Lafuente, Estefanía Martín, Rosa Patricia Arias-Llorente, Pilar Carretero, José Luis Gallo-Vallejo, Francisca Romero-Narbona, M. A. Salmerón-Ruiz, Clara Alonso-Diaz, Rafael Maese-Heredia, Lucas Cerrillos, Ana María Fernández-Alonso, Carmen Camarena, Josefa Aguayo, Miguel Sánchez-Forte, Manuel Rodríguez-Maresca, Alfredo Pérez-Rivilla, Rosa Quiles-Pérez, Paloma Muñoz de Rueda, Manuela Expósito-Ruiz, Federico García, Fernando García, Javier Salmerón

**Affiliations:** 1 Hospital Universitario San Cecilio, Granada, Spain; 2 CIBER de Enfermedades Hepáticas y Digestivas (CIBEREHD), CIBER, Madrid, Spain; 3 Universidad de Granada, Granada, Spain; 4 Instituto de Investigación Biosanitaria Ibs.GRANADA, Granada, Spain; 5 Hospital Universitario La Paz, Madrid, Spain; 6 Hospital Universitario Virgen de las Nieves, Granada, Spain; 7 Hospital Universitario Central de Asturias, Oviedo, Spain; 8 Hospital Universitario Virgen de la Victoria, Málaga, Spain; 9 Hospital Universitario Doce de Octubre, Madrid, Spain; 10 RED SAMID (ISCIII ref. RD/16/0022), Spain; 11 Hospital Universitario Virgen del Rocío, Sevilla, Spain; 12 Hospital Universitario Torrecárdenas, Almería, Spain; 13 Unidad de Apoyo a la Investigación, Hospital Universitario Virgen de las Nieves, Granada, Spain; 14 Fundación para la Investigación Biosanitaria de Andalucía Oriental (FIBAO), Granada, Spain; 15 RED de SIDA (ISCIII ref. RD/16/0025/0040), Spain; University of the Witwatersrand, SOUTH AFRICA

## Abstract

**Background & aim:**

Worldwide, measures are being implemented to eradicate hepatitis B (HBV) and C (HCV) viruses, which can be transmitted from the mother during childbirth. This study aims to determine the prevalence of HBV and HCV in pregnant women in Spain, focusing on country of origin, epidemiological factors and risk of vertical transmission (VT).

**Methodology:**

Multicentre open-cohort study performed during 2015. HBV prevalence was determined in 21870 pregnant women and HCV prevalence in 7659 pregnant women. Epidemiological and risk factors for VT were analysed in positive women and differences between HBV and HCV cases were studied.

**Results:**

HBV prevalence was 0.42% (91/21870) and HCV prevalence was 0.26% (20/7659). Of the women with HBV, 65.7% (44/67) were migrants. The HBV transmission route to the mother was unknown in 40.3% of cases (27/67) and VT in 31.3% (21/67). Among risk factors for VT, 67.7% (42/62) of the women had viraemia and 14.5% (9/62) tested HBeAg-positive. All of the neonates born to HBV-positive mothers received immunoprophylaxis, and none contracted infection by VT. In 80% (16/20) of the women with HCV, the transmission route was parenteral, and nine were intravenous drug users. Viraemia was present in 40% (8/20) of the women and 10% (2/20) were HIV-coinfected. No children were infected. Women with HCV were less likely than women with HBV to breastfeed their child (65% vs. 86%).

**Conclusions:**

The prevalences obtained in our study of pregnant women are lower than those previously documented for the general population. Among the women with HBV, the majority were migrants and had a maternal family history of infection, while among those with HCV, the most common factor was intravenous drug use. Despite the risk factors observed for VT, none of the children were infected. Proper immunoprophylaxis is essential to prevent VT in children born to HBV-positive women.

## Introduction

According to the World Health Organization (WHO) [1], chronic infection by hepatitis B virus (HBV) affects 257 million people. It is particularly prevalent in sub-Saharan Africa and in East Asia, affecting 5–10% of all adults, while in North America, less than 1% are infected. The estimated prevalence is less than 5% in eastern Europe, 1.5% in northern Europe, 2% in southern Europe and 1% in western Europe. However, these values are probably underestimates because, among other reasons, the data are obtained from blood donors [2]. Moreover, the situation in Europe may be adversely affected by changing patterns of migration [3,4]. According to a systematic review conducted by the European Centre for Disease Prevention and Control (ECDC) [5] based on articles published from 2005 to 2015, the prevalence in Spain was around 0.8% (0.6–1.1) and according to the latest report available [3] it was 0.66% (0.34–0.97).

The WHO has reported that 71 million people are infected with hepatitis C virus (HCV) [1] and that the most severely affected regions are Europe and the Eastern Mediterranean (2.3%). In Europe, the prevalence of HCV antibodies (anti-HCV) is estimated at 1.1% (95%CI 0.9–1.4) [5]. In Spain, the prevalence of antibodies is 1.7% and that of viraemia (HCV RNA positive) is 1.2% [6]. In the Ethon Cohort [7], the prevalence of anti-HCV positive (anti-HCV+ve) was 1.23% and only 0.32% had viraemia. However, this prevalence might be affected by changes in patterns of migration in Europe, especially in Spain [8].

In Spain, the prevalence of HBV infection (surface antigen positive) in pregnant women ranges from 0.1% to 4.4% [5,9,10]. The vertical transmission (VT) of HBV continues to be one of the main routes of infection worldwide [11], especially in areas where it is endemic [12]. High viral load (VL), which mainly affects HBeAg-positive (HBeAg+ve) women, is the most important risk factor, although women who are HBeAg-negative (HBeAg-ve) and who present high VL are also at risk of transmission [13]. In this population, 90% chronicity of infected newborns has been reported [14].

The prevalence of anti-HCV in pregnant women in Spain is estimated to be 0.5–1.4% [9,15,16], and the prevalence of viraemia 42–72%. The rate of VT among women with HCV is low, 1–8% in non-coinfected mothers and around 20% in those coinfected with HIV. However, 90% of infected children acquire the virus by VT. The factors related to the VT of HCV are VL and HIV coinfection [17–19]. No other factors related to childbirth, epidemiology or breastfeeding have been associated with VT.

Screening for HBV in pregnancy is routinely recommended, as immunoprophylaxis against VT is feasible [12,20]. According to 2015 data, generalised immunoprophylaxis at birth has reduced the proportion of chronically-infected children aged under five years from 4.7% to 1.3% [1]. Nevertheless, up to 10% of newborns whose mothers present high VL become infected [14,21]. Accordingly, it is recommended that in HBV+ve women, VL should be assessed at 24 weeks of pregnancy and antiviral treatment administered if the VL is high. On the other hand, the determination of HCV in pregnancy is only recommended for at-risk populations such as intravenous drug users, women with high-risk sexual practices, women with HIV coinfection and women born in countries with high endemicity for HCV. In consequence, the prevalence reported in the literature may not correspond to reality. Prophylactic and therapeutic measures to eradicate HBV and HCV are currently being implemented. Children infected by VT should be included in this programme, but screening during pregnancy for HCV is not yet universal, and therefore some children who acquire the virus through VT will remain undiagnosed.

In view of these considerations, the main aim of this study is to determine the prevalence of HBV and HCV in pregnant women in Spain, taking into account their country of origin, epidemiological factors and the risk of VT.

## Material and method

### Patients

To determine the prevalence of HBV and HCV among a population of pregnant women, a multicentre prospective open-label cohort study was carried out from January to December 2015. The following hospitals took part: Hospital Universitario 12 de Octubre Madrid (HU12O), Hospital Universitario La Paz Madrid (HULP), Hospital Universitario San Cecilio Granada (HUSC), Hospital Universitario Virgen de las Nieves Granada (HUVN), Hospital Universitario Central de Asturias Oviedo (HUCA), Hospital Universitario Torrecárdenas Almería (HUT), Hospital Universitario Virgen del Rocío Sevilla (HUVR) and Hospital Universitario Virgen de la Victoria Málaga (HUVV).

**Fig 1:**
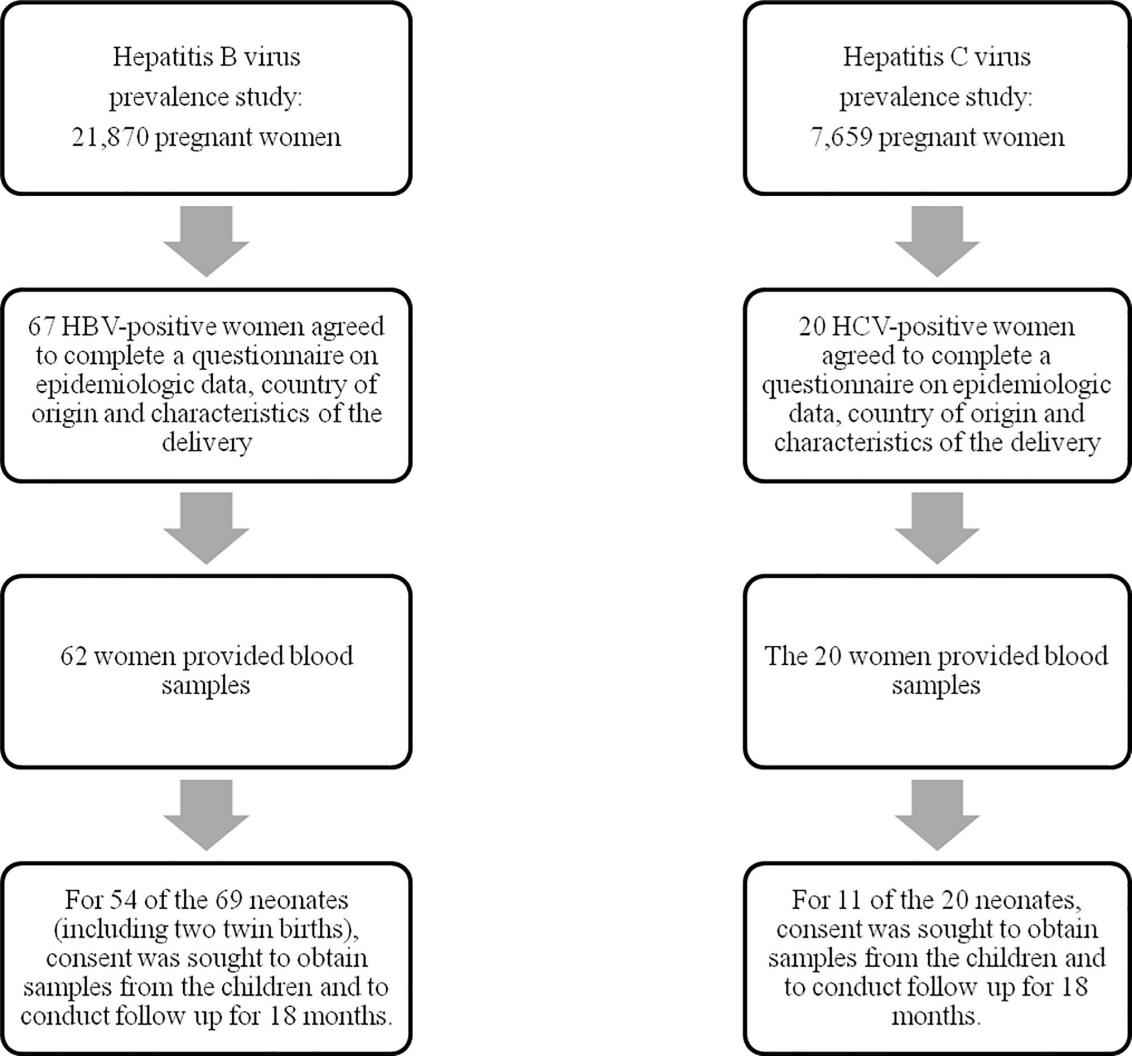
Flow chart for selection of participants. This figure shows the flow chart of pregnant women recruitment for prevalence, epidemiological and risk factors for vertical transmission studies.

The population analysed in the HBV prevalence study consisted of 21870 pregnant women, whose data were provided by the researchers at each hospital, from an anonymised database managed by its Microbiology Service, which compiles and manages these data as usual clinical practice in pregnancy. After the delivery, the mothers found to be HBV-positive (HBV+ve) were invited to participate in the study, by completing the epidemiological survey, stating their country of origin and providing details of the delivery. In every case, the mother’s informed consent was obtained. In addition, the donation of blood samples from each mother and child was requested, together with consent for medical follow-up of the neonate for 18 months. Fig 1 shows the flow chart for the recruitment of these participants.

In Spain, the determination of HCV does not form part of usual clinical practice, and so informed consent in this respect was requested and obtained in every case included in this study. Accordingly, tests for HCV were performed on 7659 women in week 12 of pregnancy, during the scheduled hospital visit for the detection of chromosomopathies. After the delivery, the mothers found to be HCV positive (HCV+ve) were invited to participate in the study, by completing the epidemiological survey, stating their country of origin and providing details of the delivery. In every case, informed consent in this respect was obtained. In addition, the donation of blood samples from the mother and child was requested, together with consent for medical follow-up of the neonate for 18 months. Fig 1 shows the flow chart for the recruitment of these participants.

The inclusion criteria were HBV surface antigen positive (HBsAg+ve) or anti-HCV+ve status confirmed by enzyme-linked immunosorbent assay (ELISA) and the provision of informed consent.

### Epidemiological study

The study variables were participant’s age, country of origin and risk factors for infection. The risk factors considered were transfusion, intravenous drug abuse (IVDA), tattoos, piercing, surgery, dental treatment, infected partner, high-risk sexual practices, history of infection in the participant’s mother or siblings and HIV coinfection, categorised as yes, no or unknown. The survey also asked whether the woman knew about the infection prior to her pregnancy. The route of transmission of infection was categorised as vertical, parenteral, sexual or unknown.

### Risk factors for vertical transmission

Antigen E (HBeAg) and antibody E (anti-HBe) were analysed for HBV, and VL was analysed for HBV and/or HCV.

The HBV+ve women were classified according to the EASL Clinical Practice Guidelines [22] as HBeAg-positive chronic infection (HBeAg+ve and DNA >10^7^ IU/mL), HBeAg-positive chronic hepatitis (HBeAg+ve and DNA 10^4^−10^7^ IU/mL), HBeAg-negative chronic infection (HBeAg-ve and DNA <2000 IU/mL) and HBeAg-negative chronic hepatitis (HBeAg-ve and DNA >2000 IU/mL).

Other information compiled for the study included data on the pregnancy and delivery, including in vitro fertilisation, antiviral treatment for HBV during pregnancy, type of delivery, gestational age, time elapsed since the breaking of the waters, weight, Apgar score of the neonate at birth and the type of lactation.

The study protocol is the same as has been described in previous studies by this research group (17–19).

### Analysis of the differences between HBV and HCV

The dependent variable was HBV+ve versus HCV+ve. The remaining variables were assumed to be independent.

### Virologic assays

The serological determination of HBsAg, anti-HCV and anti-HIV was carried out by commercial ELISA, which is routinely used in the laboratories of each of the participating hospitals.

The VL of HBV and/or HCV was determined by COBAS TaqMan (cut-off <12 IU/mL, <15 IU/mL. Roche Diagnostics, respectively), distinguishing between not detected, below cut-off but not quantifiable and greater than cut-off and quantifiable. In the HBV+ve women, HBeAg and anti-HBe were also determined.

### Statistical analysis

In our statistical analysis, the quantitative variables are described by the mean and the standard deviation, or in cases of non-normal distribution, by the median and the interquartile range. The qualitative variables are presented as absolute and relative frequencies. The corresponding prevalences and 95% confidence intervals were also calculated. The inter-group differences were determined by bivariate analysis, using Pearson’s chi-square test or Fisher’s exact test for the qualitative variables and Student’s t test or the Mann-Whitney U test for the continuous ones. The normality of the data distribution was examined by the Kolmogorov-Smirnov test. A p-value <0.05 was considered significant. All data analyses were performed using IBM SPSS 19 statistical software.

### Ethical considerations

This study was carried out in accordance with the ethical guidelines of the Declaration of Helsinki of 1975, revised in 2013. All participants gave written and verbal informed consent. The study protocol was firstly approved by Comité Ético de Investigación Provincial de Granada (Ethics Committee of the Principal Investigator’s hospital) and later by the Ethics Committee at each participating hospital. The relevant provisions of Spanish data protection legislation were respected in all phases of the study.

## Results

### Prevalence of HBV and HCV in pregnancy

The prospective cohort for the HBV study consisted of 21870 pregnant women, of whom 91 were HBsAg+ve, with a prevalence of 0.42% (95% CI 0.33–0.50). Table 1 shows the distribution among the participating hospitals. The cohort for the HCV study contained 7659 women, of whom 20 were anti-HCV+ve, with a prevalence of 0.26% (95% CI 0.15–0.38). The prevalence of HBV differed significantly among the hospitals (p = 0.013), lowest at HUCA and highest at HULP, HUT and HUVR. On the other hand, the inter-hospital differences for anti-HCV prevalence were not significant (p = 0.52).

**Table 1:** Seroprevalence of HBV and HCV among pregnant women at each hospital.

**HBV**	Positive cases	Women (n)	Prevalence (%)	95%CI
HU12O^a^	18	4224	0.42	0.22–0.62
HULP^b^	17	2901	0.59	0.31–0.86
HUSC^c^ –HUVN^d^	9	2262	0.39	0.14–0.66
HUCA^e^	1	2362	0.04	0.01–0.12
HUT^f^	13	2037	0.64	0.29–0.98
HUVR^g^	29	5903	0.49	0.32–0.67
HUVV^h^	4	2181	0.18	0.004–0.362
**Total**	**91**	**21870**	**0.42**	**0.33–0.50**
**HCV**	Positive cases	Women (n)	Prevalence (%)	95%CI
HULP^b^	7	2901	0.24	0.06–0.42
HUSC^c^ –HUVN^d^	2	903	0.22	0.01–0.53
HUCA^e^	5	1713	0.29	0.04–0.55
HUT^f^	3	1671	0.18	0.01–0.38
HUVV^h^	3	471	0.64	0.01–1.34
**Total**	**20**	**7659**	**0.26**	**0.15–0.38**

^a^HU12O: Hospital Universitario 12 de Octubre, Madrid.

^b^HULP: Hospital Universitario La Paz, Madrid.

^c^HUSC: Hospital Universitario San Cecilio, Granada.

^d^HUVN: Hospital Universitario Virgen de las Nieves, Granada.

^e^HUCA: Hospital Universitario Central de Asturias, Oviedo.

^f^HUT: Hospital Universitario Torrecárdenas, Almería.

^g^HUVR: Hospital Universitario Virgen del Rocío, Sevilla.

^h^HUVV: Hospital Universitario Virgen de la Victoria, Málaga.

### Epidemiology and risk factors for vertical transmission in HBV-positive women

**Fig 2:**
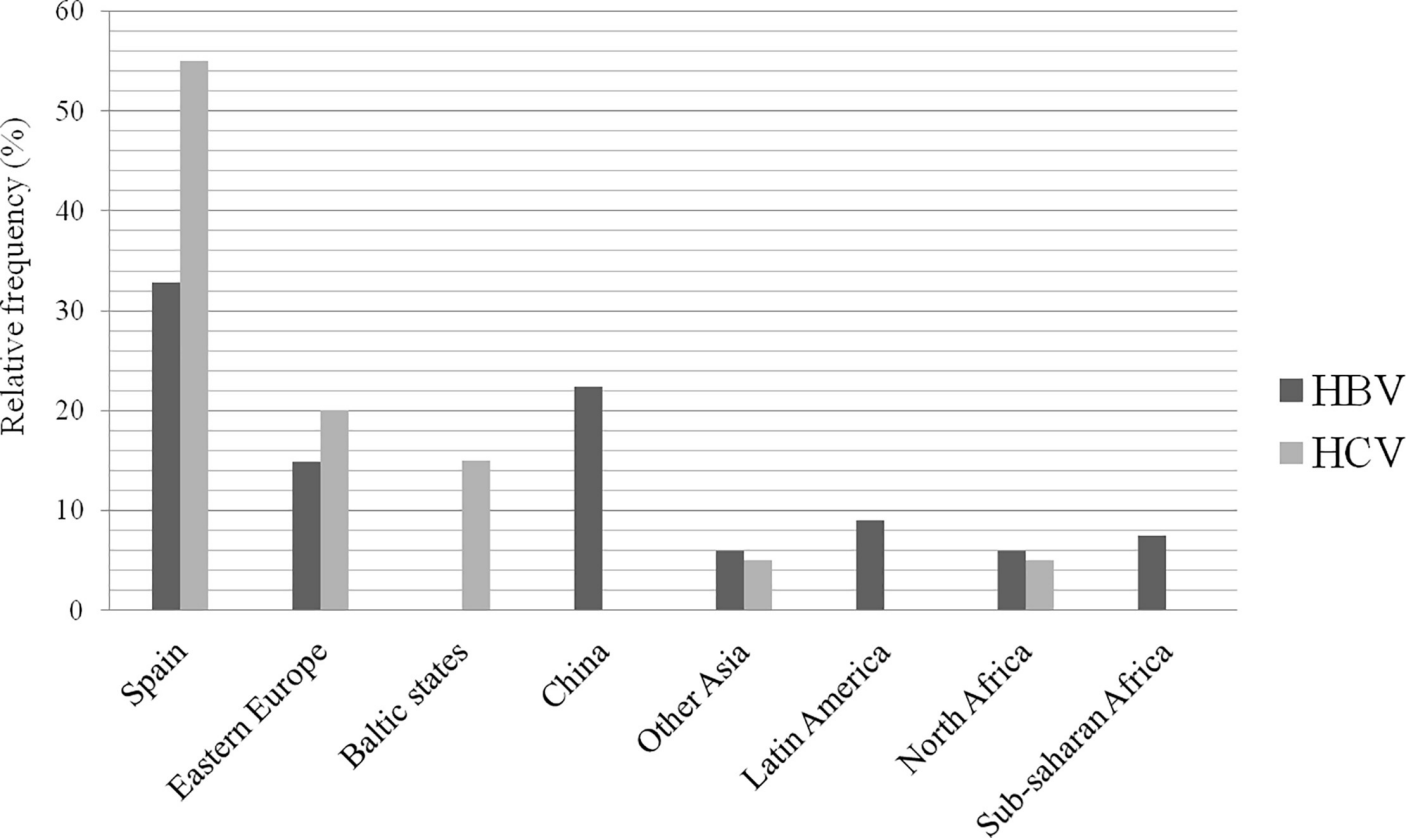
Geographic origin of pregnant women with HBV or HCV infection. This figure shows the geographic origin of the pregnant women with HBV or HCV infection who participated in the epidemiological study, expressed in relative frequency.

Of the 67 women included in the study, 31 (46.3%) were aware of the infection prior to pregnancy. None were coinfected with HCV, and there was one case (1.5%) of coinfection with HIV. By country of birth, 22 (32.8%) were Spanish, followed in frequency by those from China and Eastern Europe (Fig 2). The risk factors for infection in these pregnant women are shown in Table 2. The route of transmission was parenteral in 14 cases (20.9%), vertical in 21 (31.3%), sexual in 3 (4.5%) and unknown in 27 (40.3%). Of the women presenting VT, 13 (61.9%) were of foreign origin.

**Table 2:** Epidemiology of pregnant women infected with HBV or HCV.

	HBV n = 67	HCV n = 20	P
**Median age of the mother** [P25-P75]	33 [28–37]	34 [32.2–35.7]	0.106
**Aware of infection**	31 (46.3%)	13 (65%)	0.128
**Co-infection with HIV**	1 (1.5%)	2 (10%)	0.125
**Country of origin**			
Spain	22 (32.8%)	11 (55%)	0.070
Other	44 (65.7%)	9 (45%)	
**Transfusion**	1 (1.5%)	3 (15%)	0.043
**IVDA**^a^	0 (0%)	9 (45%)	<0.001
**Tattoo**	6 (9%)	2 (10%)	1
**Piercing**	3 (4.5%)	1 (5%)	1
**Surgery**	13 (19.4%)	6 (30%)	0.383
**Dental treatment**	4 (6%)	1 (5%)	1
**Infected partner**	3 (4.5%)	3 (15%)	0.143
**High-risk sexual practices**	3 (4.5%)	3 (15%)	0.148
**Family history**^b^	21 (31.3%)	0 (0%)	0.001
**Unknown route of infection**	27 (40.3%)	4 (20%)	0.066

^a^IVDA: Intravenous drug abuse.

^b^Family history: previous infection in the mother or siblings.

Of the 62 HBsAg+ve women who provided samples, 42 (67.7%) had viraemia. Nine (14.5%) were HBeAg+ve and one (with a twin pregnancy) had chronic infection with a VL of 2070 IU/mL at the moment of delivery. Of the eight women who had HBeAg+ve chronic infection, six were treated during pregnancy: three achieved undetectable VL before delivery and in the other three the maximum VL was 1448 IU/mL. The two HBeAg+ve women who were not treated had a VL >10^8^ IU/mL. Two of the HBeAg+ve women were Spanish, five were Chinese, one was from Central America and one was from sub-Saharan Africa. The ten neonates were monitored after birth, and none became infected. Of the 53 (85.5%) women who were HBeAg-ve, 40 had chronic infection and 13 had chronic hepatitis.

None of the pregnancies was achieved by in vitro fertilisation. The birth was spontaneous in 47 (72.3%) cases, by caesarean section in 13 (20%) (two of which were scheduled) and by instrumental delivery in five (7.7%). The data for the 69 neonates are summarised in Table 3. Only one of the low-weight children had a maternal history of antiviral treatment. Of the 30 neonates in whom the VL was analysed at birth, two were HBV+ve, although later tests were negative. All of the neonates born to HBsAg+ve women received immunoprophylaxis during the first twelve hours of life, for an average of 3.4 ± 3 hours. The ten children born to HBeAg+ve women received immunoprophylaxis in the first three hours of life. None of the 54 children who completed the follow-up became infected.

**Table 3:** Characteristics of the neonates born to HBV or HCV-positive mothers.

	HBV n = 69	HCV n = 20	P
**Premature**^a^	8 (11.6%)	4 (20%)	0.265
**Weight** (g) Mean ± standard deviation	3194.7 ± 681	2998.8 ± 582	0.248
**Time elapsed since breaking of the waters (h)** Mean ± standard deviation	7 ± 8	12 ± 17	0.212
**Apgar score** Median [P25-P75]	9 [5–10]	9 [6–10]	0.834
**Breastfeeding**	55 (86%)	13 (65%)	0.044

^a^Premature: gestational age <37 weeks.

### Epidemiology and risk factors for vertical transmission in HCV-positive women

Thirteen women (65%) were previously aware of their HCV infection. None were coinfected with HBV and there were two cases (10%) of coinfection with HIV. By country of origin, the majority of women were from Spain (11 cases, 55%) and Eastern Europe (four cases, 20%) (Fig 2). The risk factors for infection are detailed in Table 2. The route of transmission was parenteral in 16 women (80%), of whom nine had a history of IVDA. The route of transmission was unknown in four cases (20%).

HCV RNA was positive (HCV-RNA+ve) in eight (40%) of the 20 women, with an average VL of 1.9x10^5^ IU/mL (4.4x10^5^–3.5x10^6^ IU/mL). The distribution of genotypes was 1b (25%), 3 (25%), 4c (12.5%) and unknown (37.5%).

None of the pregnancies was achieved by in vitro fertilisation. Delivery was spontaneous in 12 cases (60%), by caesarean section in four (20%) and by instrumental delivery in four (20%). The data for the 20 neonates are summarised in Table 3. Five children of the eight women with viraemia completed the follow up and none became infected.

### Analysis of the differences between HBV and HCV-positive pregnant women

As can be seen in Table 2, 65.7% (44/67) of the women with HBV were foreign born, versus only 45% (9/20) of the women with HCV. Nevertheless, the difference was not statistically significant (p = 0.07). A similar pattern was observed for the unknown route of maternal infection, which was more frequent among the women with HBV than those with HCV (40.3% vs. 20%; p = 0.066). VT as the mechanism of infection was more frequent for the HBV+ve women (31.3% vs. 0%; p = 0.001), while IVDA was more frequent in the HCV group (45% vs. 0%; p<0.001). Previous blood transfusion was also significantly higher in the HCV group (15% vs. 1.5%; p = 0.043).

There were no statistically significant differences among the neonates for any of the variables analysed, except that a significantly lower proportion of the women with HCV breastfed their children (65% vs. 86%; p = 0.044) (Table 3).

## Discussion

In our study population, the prevalence of HBV was 0.42% (91/21870) and that of HCV was 0.26% (20/7659). The HBV+ve women were more likely to be foreign born than those with HCV. The most frequent route of transmission of HBV was of unknown origin, while among those of known origin, VT was most commonly observed. Regarding the risk factors for VT, 67.7% of the women presenting HBV had viraemia and 14.5% were HBeAg+ve. Immunoprophylaxis was administered correctly to all the neonates born to HBV+ve mothers. Among the HCV+ve mothers, the parenteral route of transmission was most common, mainly due to IVDA. In the HCV group, 40% had viraemia and 10% were co-infected with HIV. The HCV+ve women were less likely than HBV+ve to breastfeed their children.

In this study, the observed prevalence of HBsAg+ve was significantly different among the participant hospitals. The highest level was obtained for the HUT in Almeria (0.64%), a hospital that serves a large migrant population from North and sub-Saharan Africa. In contrast, the data for anti-HCV prevalence were fairly consistent among the participant hospitals, with the sole exception of the HUVV in Malaga, where the prevalence was higher, although in this case the sample size was very small.

In the general population in Spain, the prevalence of HBV is 0.66% (0.34–0.97) [3], which is higher than the prevalence we found in pregnant women. In a previous study conducted in 1986–89 [23] at the HUSC in Granada, based on a population of 4450 pregnant women, the prevalence of HBV was 1.53% (95% CI 1.14–1.92). Thirty years later, according to our findings, the prevalence in this city has fallen to 0.39%. For comparison, a 2009 study by Salleras *et al*. [24], with a population of pregnant women in Catalonia, and which has been taken as a benchmark for Spain by the ECDC [5], recorded a very low prevalence, of 0.1%.

Our findings show that 65.7% of the participants who were HBV+ve were foreign born, with a particularly strong presence of women from China and eastern Europe, which corroborates the pattern reported previously [25,26]. In a study conducted in Madrid [26], the highest rate of seropositivity was found among women from Romania and other Eastern European countries (23.7%), followed by those from China (20.3%) and sub-Saharan Africa (18.6%). In recent years, the prevalence of HBV in Spain has tended to decrease, and if the population considered excluded those of foreign origin, the prevalence would probably be even lower.

In most of the cases analysed in our study, the route of transmission of HBV infection was unknown, while the most common of the known routes was maternal family history of HBV infection (31.3%), although some women presented several risk factors for infection. A large majority of the women (67.7%) had viraemia and therefore were potential transmitters of infection. 14.5% were HBeAg+ve, which increased the risk of transmission still further. However, only six of these women received antiviral treatment during their pregnancy, which shows that not all hospitals follow clinical practice guidelines in this respect [22]. A previous study carried out in Granada with 4169 pregnant women [25] reported that HBV was present in 27, of whom only one (a woman of Asian origin) was HBeAg+ve.

All of the 69 neonates born to HBV+ve mothers received immunoprophylaxis during the first twelve hours of life, and the ten children born to HBeAg+ve women received immunoprophylaxis in the first three hours of life. None of the 54 neonates who completed the follow up became infected. This includes the two children of HBeAg+ve women with high VL who were untreated, which highlights the importance of correctly administering immunoprophylaxis to neonates at an early stage of life.

In our study, the prevalence of HCV was 0.26%, and only 40% of these cases were HCV-RNA+ve. On the other hand, previous studies in the general population have found a higher prevalence, of 1.7% (0.4–2.6), with HCV-RNA+ve in 68.6% of these cases [27]. Nevertheless, research has shown that the prevalence of HCV is lower among women than in the general population. In a study conducted in the United States [28], the prevalence in men was 1.56% versus 0.75% in women. In another study, also conducted in the USA [29], with 87924 pregnant women, the prevalence of HCV was 1.2%. In Navarre (Spain), a study of 7314 pre-surgical patients [30] revealed a prevalence of 0.94%, with higher values for men than for women (1.25% vs. 0.62%, p = 0.0049). Finally, a study carried out in 1993–95 with 3003 pregnant women at the HUSC in Granada [19] reported that only 19 were HCV+ve (0.63%) while 14 (74%) presented viraemia. Thus, the value of 0.26% obtained in the present study is much lower than these previous findings.

Among our study population, 55% of the women with HCV were Spanish, followed in frequency by those from Eastern Europe (20%), which is in line with previous findings [31]. According to earlier research [27], 80% of the cases recorded worldwide with positive viraemia are located in 31 countries, one of which is Spain, but over half of the infections correspond to just six countries: China, Pakistan, Nigeria, Egypt, India and Russia. Parenteral transmission is the most common route of infection, and most cases involve a history of IVDA, although in 20% of cases the origin of the infection is not known. This knowledge gap could justify the introduction of HCV screening in pregnancy. Similar findings have been reported in other studies [29,32,33]. Infection is significantly more frequent in people aged under 30 years, those who are of European descent, especially those from Eastern Europe, and those with a history of IVDA. The most frequent genotypes recorded in our study were 1b and 3, followed by 4, which is consistent with previous research findings [33]. Two women (10%) had co-infection by HIV, which increases the probability of HCV being transmitted to the child [17].

Among the children of women with HCV infection, 20% were premature, a value that is higher than in the general population. Moreover, only 65% initiated breastfeeding, which is well below the figure for children of HBV+ve mothers (86%) and the general population. These findings may be more influenced by the history of IVDA than by the HCV *per se* [34]. This may also be the reason why not all the children of mothers with viraemia completed the follow up.

Among the specific characteristics differentiating the HBV+ve women were the predominance of infection by VT and the larger proportion who breastfed their children. In addition, more were foreign born, compared to the women with HCV. In the HCV group, the main distinguishing features observed were blood transfusion and a history of IVDA.

The main limitation of this study concerned the determination of HCV in the pregnant women, since screening for this condition is not universal in Spain during pregnancy, and therefore signed informed consent was required. For this reason, the sample size for our analysis of HCV prevalence was lower than that for HBV. Furthermore, some of the Spanish hospitals that were invited declined to participate. Another limitation concerned the follow-up of the children of seropositive mothers; many of these children were adopted after birth and so their place of residence changed.

In summary, the prevalences of HBV and HCV that we report are lower than those documented previously. A significant number of the women with HBV were foreign born and/or had a maternal family history of infection. Among those with HCV, many had a history of IVDA, which probably influenced the fact that these women were less likely to complete follow up and less likely to breastfeed their children. Over half of the women with HBV had viraemia, and therefore were potential transmitters of the infection to the neonate. Therefore, the provision of appropriate immunoprophylaxis (including immunoglobulin therapy and vaccination) is important in neonates born to HBV+ve mothers. However, among the women with HCV, despite the presence of viraemia and coinfection with HIV, there was no transmission to the children.

## References

[1] World Health Organization. Global Hepatitis Report 2017. [Internet] Geneva: World Health Organization; 2017 Available from: http://www.who.int/hepatitis/publications/global-hepatitis-report2017/en/.

[2] Pimpin L, Cortez-Pinto H, Negro F, Corbould E, Lazarus JV, Webber L, et al Burden of liver disease in Europe: epidemiology and analysis of risk factors to identify prevention policies. Journal of Hepatology 2018;69:718–35. 10.1016/j.jhep.2018.05.011 29777749

[3] Ahmad AA, Falla AM, Duffell E, Noori T, Bechini A, Reintjes R, et al Estimating the scale of chronic hepatitis B virus infection among migrants in EU/EEA countries. BMC Infect Dis 2018;18(1):34–48. 10.1186/s12879-017-2921-8 29325525 PMC5765695

[4] Ortiz E, Scanlon B, Mullens A, Durham J. Effectiveness of interventions for hepatitis B and C: a systematic review of vaccination, ccreening, health promotion and linkage to care within higher income countries. J Community Health 2019. [Epub ahead of print]. 10.1007/s10900-019-00699-6 31332639

[5] Hofstraat SHI, Falla AM, Duffell EF, Hahné SJM, Amato-Gauci AJ, Veldhuijzen IK, et al Current prevalence of chronic hepatitis B and C virus infection in the general population, blood donors and pregnant women in the EU/EEA: a systematic review. Epidemiol Infect 2017;145(14):2873–85. 10.1017/S0950268817001947 28891457 PMC5647665

[6] Ministerio de Sanidad, Servicios Sociales e Igualdad. Plan estratégico para el abordaje de la hepatitis C en el Sistema Nacional de Salud. [Internet] Madrid: Ministerio de Sanidad, Servicios Sociales e Igualdad; 2015 Available from: https://www.mscbs.gob.es/ciudadanos/enfLesiones/enfTransmisibles/docs/plan_estrategico_hepatitis_C.pdf.

[7] Cuadrado A, Perello C, Llerena S, Escudero-García D, Gómez M, Estébanez A, et al Design and cost effectiveness of a hepatitis C virus elimination strategy based on an updated epidemiological study (ETHON cohort). Poster presented at European Association for the Study of the Liver, The International Liver Congress, Paris. J Hepatol 2018;68 (Suppl 1):S164.

[8] Falla AM, Ahmad AA, Duffell E, Noori T, Veldhuijzen IK. Estimating the scale of chronic hepatitis C virus infection in the EU/EEA: a focus on migrants from anti-HCV endemic countries. BMC Infect Dis 2018;18(1):42–55. 10.1186/s12879-017-2908-5 29338702 PMC5771208

[9] Ruiz-Extremera A, López-Garrido MA, Barranco E, Quintero MD, Ocete-Hita E, Muñoz de Rueda P, et al Activity of hepatic enzymes from week sixteen of pregnancy. Am J Obstet Gynecol 2005;193(6):2010–6. 10.1016/j.ajog.2005.04.045 16325605

[10] Aristegui Fernández J, Diez-Domingo J, Mares Bermudez J, Martinon Torres F. Vacunación frente a la hepatitis B. Impacto de los programas de vacunación tras 20 años de su utilización en España. ¿Es tiempo de cambios? Enferm Infecc Microbiol Clin 2015;33(2):113–8. 10.1016/j.eimc.2014.12.010 25620127

[11] Dionne-Odom J, Tita AT, Silverman NS. Hepatitis B in pregnancy screening, treatment, and prevention of vertical transmission. Society for Maternal-Fetal Medicine (SMFM). Am J Obstet Gynecol 2016;214:6–14. 10.1016/j.ajog.2015.09.100 26454123

[12] Wen WH, Lai MW, Chang MH. A review of strategies to prevent mother-to-infant transmission of hepatitis B virus infection. Expert Rev Gastroenterol Hepatol 2016;10(3):317–30. 10.1586/17474124.2016.1120667 26566769

[13] Bleich LM, Swenson ES. Prevention of neonatal hepatitis B virus transmission. J Clin Gastroenterol 2014;48(9):765–72. 10.1097/MCG.0000000000000115 24667588

[14] Jonas MM. Hepatitis B and pregnancy: an underestimated issue. Liver Int 2009;29(Suppl 1):133–9. 10.1111/j.1478-3231.2008.01933.x 19207977

[15] Muñoz-Almagro C, Juncosa T, Fortuny C, Guillén JJ, González-Cuevas A, Latorre C. Prevalence of hepatitis C virus in pregnant women and vertical transmission. Med Clin 2002;118(12):452–4. 10.1016/s0025-7753(02)72417-2 11958762

[16] Benova L, Mohamoud YA, Calvert C, Abu-Raddad LJ. Vertical transmission of hepatitis C virus: systematic review and meta-analysis. Clin Infect Dis 2014;59(6):765–73. 10.1093/cid/ciu447 24928290 PMC4144266

[17] Ruiz-Extremera A, Muñoz-Gámez JA, Salmerón-Ruiz MA, de Rueda PM, Quiles-Pérez R, Gila-Medina A, et al Genetic variation in interleukin 28B with respect to vertical transmission of hepatitis C virus and spontaneous clearance in HCV-infected children. Hepatology 2011;53(6):1830–8. 10.1002/hep.24298 21413051

[18] Ruiz-Extremera A, Salmerón J, Torres C, De Rueda PM, Giménez F, Robles C, et al Follow-up of transmission of hepatitis C to babies of human immunodeficiency virus-negative women: the role of breast-feeding in transmission. Pediatr Infect Dis J 2000;19(6):511–6. 10.1097/00006454-200006000-00004 10877164

[19] Salmerón J, Giménez F, Torres C, Ros R, Palacios A, Quintero D, et al Epidemiology and prevalence of seropositivity for hepatitis C virus in pregnant women in Granada. Rev Esp Enferm Dig 1998;90(12):841–50. 9973846

[20] Yi P, Chen R, Huang Y, Zhou RR, Fan XG. Management of mother-to-child transmission of hepatitis B virus: Propositions and challenges. J Clin Virol 2016;77:32–9. 10.1016/j.jcv.2016.02.003 26895227

[21] Pan CQ, Duan ZP, Bhamidimarri KR, Zou HB, Liang XF, Li J, et al An algorithm for risk assessment and intervention of mother to child transmission of hepatitis B virus. Clin Gastroenterol Hepatol 2012;10(5):452–9. 10.1016/j.cgh.2011.10.041 22079509

[22] European Association for the Study of the Liver. EASL 2017 Clinical Practice Guidelines on the management of hepatitis B virus infection. J Hepatol 2017;67(2):370–98. 10.1016/j.jhep.2017.03.021 28427875

[23] Del Castillo G, Ruiz-Extremera A, López F, Tomás J, Jiménez F, Salmerón FJ. Prevalencia del VHB en mujeres gestantes. Inmunoprofilaxis. Rev Soc Andal Pat Digest 1989;12:447–50.

[24] Salleras L, Domínguez A, Bruguera M, Plans P, Espuñes J, Costa J, et al Seroepidemiology of hepatitis B virus infection in pregnant women in Catalonia (Spain). J Clin Virol 2009;44(4):329–32. 10.1016/j.jcv.2009.01.002 19230752

[25] Sampedro A, Mazuelas P, Rodríguez-Granger J, Torres E, Puertas A, Navarro JM. Marcadores serológicos en gestantes inmigrantes y autóctonas en Granada. Enferm Infecc Microbiol Clin 2010;28(10):694–97. 10.1016/j.eimc.2010.04.007 20961669

[26] López-Fabal F, Gómez-Garcés JL. Marcadores serológicos de gestantes españolas e inmigrantes en un área del sur de Madrid durante el periodo 2007–2010. Rev Esp Quimioter 2013;26(2):108–11. 23817647

[27] Gower E, Estes C, Blach S, Razavi-Shearer K, Razavi H. Global epidemiology and genotype distribution of the hepatitis C virus infection. J Hepatol 2014;61(S):45–57. 10.1016/j.jhep.2014.07.027 25086286

[28] Hall EW, Rosenberg ES, Sullivan PS. Estimates of state-level chronic hepatitis C virus infection, stratified by race and sex, United States, 2010. BMC Infect Dis 2018;18:224–38. 10.1186/s12879-018-3133-6 29769036 PMC5956841

[29] Chappell C, Hillier SL, Crowe D, Meyn LA, Bogen DL, Krans EE. Hepatitis C virus screening among children exposed during pregnancy. Pediatrics 2018;141(6):1–14. 10.1542/peds.2017-3273 PMC5984711 29720535

[30] Aguinaga A, Díaz González J, Pérez García A, Barrado L, Martínez Baz I, Casado I, et al The prevalence of diagnosed and undiagnosed hepatitis C virus infection in Navarra, Spain, 2014–2016. Enferm Infecc Microbiol Clin 2018;36(6):325–31. 10.1016/j.eimc.2016.12.008 28110858

[31] Ramos JM, Masiá M, Padilla S, Escolano C, Bernal E, Gutiérrez F. Enfermedades importadas y no importadas en la población inmigrante. Una década de experiencia desde una unidad de enfermedades infecciosas. Enferm Infecc Microbiol Clin 2011;29(3):185–92. 10.1016/j.eimc.2010.11.011 21349607

[32] Alonso López S, Agudo Fernández S, García del Val A, Martínez Abad M, López Hermosa Seseña P, Izquierdo MJ, et al Seroprevalencia de hepatitis C en población con factores de riesgo del suroeste de la Comunidad de Madrid. Gastroenterol Hepatol 2016;39(10):656–62. 10.1016/j.gastrohep.2016.05.002 27417563

[33] Acero Fernández D, Ferri Iglesias MJ, Buxó Pujolràs M, López Nuñez C, Serra Matamala I, Queralt Molé X. Changes in the epidemiology and distribution of the hepatitis C virus genotypes in North-Eastern Spain over the last 35 years. Gastroenterol Hepatol 2018;41(1):2–11. 10.1016/j.gastrohep.2017.09.004 29150360

[34] Pergam SA, Wang CC, Gardella CM, Sandison TG, Phipps WT, Hawes SE. Pregnancy complications associated with hepatitis C: data from a 2003–2005 Washington state birth cohort. Am J Obstet Gynecol 2008;199(Suppl 1):38–51. 18486089 10.1016/j.ajog.2008.03.052 PMC2517631

