# Supplementary material for: Seroprevalence and epidemiology of hepatitis B and C viruses in pregnant women in Spain. Risk factors for vertical transmission
Source: PLoS One. 2020 May 21;15(5):e0233528. doi: 10.1371/journal.pone.0233528 (PMC7241747; doi:10.1371/journal.pone.0233528)
Supplement: Attachment — Submitted filename: Rebuttal letter version 3.doc [file pone.0233528.s001.doc]

María del Mar Díaz Alcázar

Hospital Universitario Clínico San Cecilio

Avenida del Conocimiento, s/n

18016 Granada (Spain)

E-mail: mmardiazalcazar@gmail.com

Joerg Heber

Editor-in-Chief

PlosOne

January 13, 2020

Dear Editor:

I am pleased to submit an original research article entitled “Seroprevalence and epidemiology of hepatitis B and C viruses in pregnant women in Spain. Risk factors for vertical transmission” by Ángeles Ruiz-Extremera, María del Mar Díaz-Alcázar, José Antonio Muñoz-Gámez, *et al*., for publication in *PlosOne*.

A previous version of this article was presented for consideration in PlosOne, but it did not meet your criteria for publication. We have carefully examined the reviewers' comments and we submit the manuscript as a new article for consideration.

We have reviewed the study design, data analysis/representation and overall manuscript write-up, so we think that it is now clearer and more concise. The answer to the major points suggested by Reviewer #1 are:

1. The study cohorts for HBV and HCV are different because the analysis of HCV does not form part of usual clinical practice. The data from HBV were provided by the researchers at each hospital, from an anonymised database managed by its Microbiology Service, which compiles and manages these data as usual clinical practice in pregnancy. The compilation of data for the HCV study did not form part of usual clinical practice, and so informed consent was requested and obtained in every case included in this study.
2. We have added a figure with the flow chart of participants through the study. We have also reviewed the methodology, so we think that now it is clearer the number of cases in the prevalence and epidemiological studies.
3. The p values in the tables are the results of the comparison between the data for HBV and HCV.
4. We did not include the genetic analyses of the viruses when we designed this study.
5. The serological status of the HBeAg and antiHBe are analyzed in the results (lines 227 – 236) and the discussion (lines 318 – 323).
6. We have reviewed the whole manuscript and have made changes though it. Moreover, our professional translator has reevaluated it completely to improve the write-up. We think that the scientific message is meaningful after reanalysis of the data.

Reviewer #2 impressions were that we tried to publish two different studies in the same paper, so we have made several changes with the main aim of better understanding. There were two cohorts in the previous article, one for the prevalence study and another one for the epidemiological study. This is why the number of HCV-positive women in the prevalence study (n=20) did not match the number of cases in the epidemiological study (n=39). This has made the methodology more difficult to understand, so our aim with the new manuscript is to make it more understandable. In the new manuscript, as it is shown in the flow chart, we only include in the epidemiological study the cases obtained from the prevalence study. Moreover, the comparison of positive women with healthy controls has also been omitted in order to simplify the methodology and make the manuscript more easily understandable. The comparison between positive cases for HBV and HCV was already present in the previous manuscript (see table 3 from the previous manuscript). With this comparison we seek to see if the characteristics of pregnant women infected by HBV and HCV are different, as seen in our results.

The answers to the major points suggested by Reviewer #2 are:

1. After many changes in the manuscript, we have also rewritten the abstract, so we think that it is now less confusing and misleading.
2. The systematic review conducted by the European Centre for Disease Prevention and Control (ECDC) took as a benchmark for Spain a study by Salleras *et al* with a population of pregnant women in Catalonia. We clarify this information in the discussion. In the introduction, we have also rewritten the goal of the study.
3. Method is the section we have changed the most, so we think that it is now better structured. We have also included a flow chart of participants through the study. We have removed in the new manuscript all the data related to the nested case-control study.
4. We have changed the tables for better understanding.
5. We have reviewed the whole bibliography to correct the mistakes.

Reviewer #2 also pointed some others minor issues we have taken into account. As previously indicated, we have removed all the data related to case-control study. In the abstract, we have eliminated the term PDA. In the limitations of the study, we clarify that some of the Spanish hospitals that were invited declined to participate, and this is the main reason why we do not include data from several regions of Spain like Basque Country, Catalonia, Galicia, Canary Islands, Balearic Islands…

We confirm that the manuscript is original. It has not been published and is not under consideration for publication elsewhere. We confirm that the manuscript has been read and approved by all named authors and we have no conflicts of interest to disclose.

We very much appreciate the suggestions, which have been very helpful in improving the manuscript. We hope that these changes to the manuscript will contribute to a better understanding of the manuscript. In any case, we are open to consideration of any further comment.

Sincerely,

María del Mar Díaz Alcázar

Corresponding author
