# Supplementary material for: Seroprevalence and epidemiology of hepatitis B and C viruses in pregnant women in Spain. Risk factors for vertical transmission
Source: PLoS One. 2020 May 21;15(5):e0233528. doi: 10.1371/journal.pone.0233528 (PMC7241747; doi:10.1371/journal.pone.0233528)
Supplement: Attachment — Submitted filename: Response to Reviewers.doc [file pone.0233528.s002.doc]

María del Mar Díaz Alcázar

Hospital Universitario Clínico San Cecilio

Avenida del Conocimiento, s/n

18016 Granada (Spain)

E-mail: mmardiazalcazar@gmail.com

Joerg Heber

Editor-in-Chief

PlosOne

April 30, 2020

Dear Editor,

First of all, we would like to thank you for the suggestions provided regarding the article entitled “Seroprevalence and epidemiology of hepatitis B and C viruses in pregnant women in Spain. Risk factors for vertical transmission” by the authors Ángeles Ruiz-Extremera, María del Mar Díaz-Alcázar, José Antonio Muñoz-Gámez, *et al*. The changes made to the manuscript are as follows:

1. We have ensured that the manuscript meets PLOS ONE's style requirements, including those for file naming. We have included a separate “Funding Statement”, having removed this information from the Acknowledgments.
2. As suggested, we have changed outmoded terms and labels, such as “Caucasian”, which has been replaced by “European descent”.
3. We have included two references from previous studies by our group, in which we used the same questionnaire for the epidemiological study.
4. We have carefully reviewed the whole manuscript and have made changes as necessary to improve the use of English, including all the changes suggested by the Editor and the reviewers. Thus:
   - “HBeAg” instead of “E antigen” or “AgHBe”.
   - “HBV” instead of “VHB”.
5. In the revised manuscript, we point out that one way in which the prevalence considered in this study may differ from that addressed previously is that we only analysed the prevalence in pregnant women.
6. We have clarified that the neonates who received immunoprophylaxis were those born to HBV-positive mothers.
7. According to the Editor, it is not clear whether there is an overlap between the pregnant women tested for HBV and those tested for HCV. The determination of HBV in pregnancy is usual clinical practice in Spain. In consequence, the study of HBV prevalence included the data from an anonymised database managed by the Microbiology Service of each participant hospital, for all pregnant women seen during the period January to December 2015. However, the determination of HCV during pregnancy does not form part of usual clinical practice in Spain, and so informed consent in this respect was requested of the pregnant women. This is why the HCV analysis was based on a smaller population.
8. With respect to ethical considerations, the prevalence of HCV was determined following the provision of informed consent for this test, by each of the women. Consent to HBV testing was not required because in Spain this is included in usual clinical practice. When delivery took place, further informed consent was obtained for the HBV and HCV studies, in order to compile epidemiological data, the mother’s country of origin and details of the delivery, to obtain blood samples from the mother and child and for the medical follow-up of the newborn.
9. The revised manuscript states that we submitted to institutional review boards a single protocol for the whole study: prevalence, epidemiology and risk factors for vertical transmission.
10. We have carried out the suggested analysis and it is now clarified that the prevalence in the participant hospitals differed significantly for HBV but not for HCV. We have included this information in the Results section. We explain in the Discussion section that the data values for the HUT in Almeria were probably higher because this hospital serves a large migrant population from North and sub-Saharan Africa.
11. We have also compared the number of cases of HBeAg-positivity between Spanish women and the immigrant populations considered. The difference is not statistically significant.
12. We have made the changes suggested in the Abstract and have limited its length to 300 words.

Our responses to the comments made by Reviewer #1 are given below, in order:

1. The manuscript has been thoroughly revised by an English-native translator. The following specific recommendations have been implemented:
   - The text “HBV prevalence was analysed in a population of 21,870 women and that of HCV, in one of 7,659.” has been replaced by “HBV prevalence was determined in 21870 pregnant women and that of HCV in 7659 pregnant women.”
   - “Chronification” has been replaced by “Chronicity”.
2. The Abstract now includes the numerical relationship for data previously represented with percentages alone.
3. It is now stated in the Discussion section that: “In the general population in Spain, the prevalence of HBV is 0.66% (0.34-0.97), which is higher than the prevalence we found in pregnant women.”
4. With respect to the importance of HBV vaccination, it is now stated in the concluding remarks that immunoprophylaxis in children born to HBV-positive women includes immunoglobulin therapy and vaccination.

Our responses to the comments made by Reviewer #2 are given below, in order:

1. In the Methodology section, we state that the study was performed from January to December 2015.
2. The manuscript has been thoroughly revised by an English-native translator. The following specific recommendations have been implemented:
   - The expression “Viraemia was present in 40% and 10% were co-infected with HIV” has been replaced by “Viraemia was present in 40% (8/20) of the women and 10% (2/20) were HIV-coinfected”.
   - The expression “In the Ethon Cohort, the reported prevalence of anti-HCV was 1.23%, but among patients with viraemia it was only 0.32%. This prevalence could be modified by changes in patterns of migration towards the European Union in general and Spain, in particular.” has been replaced by “In the Ethon Cohort, the prevalence of anti-HCV was 1.23% and only 0.32% had viraemia. However, this prevalence might be affected by changes in patterns of migration in Europe, especially in Spain.”
   - The expression “those presenting HIV coinfection or pregnant women from countries where HCV is endemic” has been replaced by “Women with HIV coinfection and women born in countries with high endemicity for HCV”
3. We have improved the labels to Figures 1 and 2 for greater clarity.

We very much appreciate the comments and suggestions made, which have been very helpful in improving the manuscript. We hope these changes are considered appropriate and that the manuscript is now suitable for publication. In any case, we will gladly consider any further comments you may have.

Yours sincerely,

María del Mar Díaz Alcázar

Corresponding author
